# Simian varicella virus infection of Chinese rhesus macaques produces ganglionic infection in the absence of rash

**DOI:** 10.1007/s13365-012-0083-4

**Published:** 2012-03-08

**Authors:** Werner J. D. Ouwendijk, Ravi Mahalingam, Vicki Traina-Dorge, Geert van Amerongen, Mary Wellish, Albert D. M. E. Osterhaus, Don Gilden, Georges M. G. M. Verjans

**Affiliations:** 1 Department of Virology, Erasmus Medical Center, Rotterdam, The Netherlands; 2 Department of Neurology, University of Colorado School of Medicine, Aurora, CO USA; 3 Department of Microbiology, University of Colorado School of Medicine, Aurora, Colorado USA; 4 Department of Microbiology, Tulane Regional Primate Research Center, Covington, Louisiana USA

**Keywords:** Simian varicella virus, Rhesus macaques, Adaptive immune response, Viremia, Ganglia, Latency

## Abstract

Varicella-zoster virus (VZV) causes varicella (chickenpox), becomes latent in ganglia along the entire neuraxis, and may reactivate to cause herpes zoster (shingles). VZV may infect ganglia via retrograde axonal transport from infected skin or through hematogenous spread. Simian varicella virus (SVV) infection of rhesus macaques provides a useful model system to study the pathogenesis of human VZV infection. To dissect the virus and host immune factors during acute SVV infection, we analyzed four SVV-seronegative Chinese rhesus macaques infected intratracheally with cell-associated 5 × 10^3^ plaque-forming units (pfu) of SVV-expressing green fluorescent protein (*n* = 2) or 5 × 10^4^ pfu of wild-type SVV (*n* = 2). All monkeys developed viremia and SVV-specific adaptive B- and T-cell immune responses, but none developed skin rash. At necropsy 21 days postinfection, SVV DNA was found in ganglia along the entire neuraxis and in viscera, and SVV RNA was found in ganglia, but not in viscera. The amount of SVV inoculum was associated with the extent of viremia and the immune response to virus. Our findings demonstrate that acute SVV infection of Chinese rhesus macaques leads to ganglionic infection by the hematogenous route and the induction of a virus-specific adaptive memory response in the absence of skin rash.

## Introduction

Varicella-zoster virus (VZV) is an exclusively human ubiquitous neurotropic alphaherpesvirus. Primary infection usually causes varicella (chickenpox), after which the virus becomes latent in neurons of sensory ganglia along the entire neuraxis. Primary VZV infection typically results in a generalized maculopapular vesicular rash, although some infected individuals never develop rash (Heininger and Seward 2006). During primary infection, virus is transported from the respiratory mucosa to sites of secondary replication and skin via VZV-infected lymphocytes (Asano et al. 1985; Ozaki et al. 1994). Virus reaches ganglia either by retrograde axonal transport from infected skin or by hematogenous spread via VZV-infected lymphocytes (Annunziato et al. 2000; Reichelt et al. 2008). Reactivation of latent VZV results from a decline in virus-specific cellular immunity (Miller 1980), mostly in elderly individuals as well as in immunocompromised organ transplant recipients and HIV + patients, resulting in zoster and multiple other serious neurological and ocular disorders (Gilden et al. 2000; Hope-Simpson 1965).

Simian varicella virus (SVV) causes varicella in non-human primates. Like VZV, SVV becomes latent in ganglionic neurons (Gray et al. 1998; Kennedy et al. 2004; Mahalingam et al. 1991) and may reactivate to produce zoster (Mahalingam et al. 2007). SVV and VZV antibodies cross-react, and open reading frames (ORFs) of the two viruses share amino acid identity ranging from 27.3% to 75.4% (Gray et al. 2001). Intrabronchial inoculation of SVV into seronegative Indian rhesus macaques produces pathological and immunological features like those seen in primary VZV infection in humans (Messaoudi et al. 2009). The aim of the present study was to dissect virus and host immune factors after primary SVV infection of Chinese rhesus macaques.

## Materials and methods

### Cells and viruses

Wild-type SVV (SVV-WT) and SVV expressing green fluorescent protein (SVV-GFP) were used. SVV-WT (Delta herpesvirus strain) was originally isolated from a naturally infected monkey (*Erythrocebus patas*; Mahalingam et al. 1992). SVV-GFP is not attenuated in vitro or in vivo (Mahalingam et al. 2001; Mahalingam et al. 1998). Low-passage SVV isolates were obtained from peripheral blood mononuclear cells (PBMC) of acutely infected African green monkeys and propagated <5 times either in a fetal rhesus macaque lung fibroblasts (DBS-FRhL-2) to generate cell-associated SVV-GFP and wild-type stocks for intratracheal inoculation or in Vero cells to produce wild-type SVV protein lysates for ELISA and functional T-cell assays (Mahalingam et al. 2001; Mahalingam et al. 1998). Protein lysates were identically prepared from mock-infected DBS-FRhL-2.

### Macaque studies

Four juvenile (3 to 4 years old) SVV-seronegative Chinese rhesus macaques were inoculated intratracheally at the bronchial bifurcation with 5 × 10^3^ plaque-forming units (pfu) of cell-associated SVV-GFP (animals 0075 and 2135) or 5 × 10^4^ pfu cell-associated SVV-WT (animals 2207 and 9021) diluted to a volume of 5 ml in phosphate-buffered saline (PBS). While the inoculation titers varied slightly, both virus titers have been shown to produce viremia and skin rash in Indian rhesus macaques (Mahalingam et al. 2010). Animals were housed in negatively pressurized hepa-filtered BSL-3 isolator cages. Before inoculation, the abdomen and back of the animals were shaved to allow careful examination for rash every other day from 1 to 21 days postinfection (dpi). Heparinized blood samples were collected at 0, 2, 5, 7, 9, 11, 13, 15, and 21 dpi. Plasma separated from the blood by centrifugation was heat-inactivated (30 min at 56°C) and stored at −20°C. PBMCs were isolated by density-gradient centrifugation for flow cytometry, DNA, and RNA isolation and functional B- and T-cell assays (see below). Animals were euthanized at 21 dpi by sedation with ketamine (20 mg/kg body weight) followed by exsanguination. Tissue samples were snap-frozen in liquid nitrogen and stored at −80°C. The study was approved by the Institutional Animal Welfare Committee and performed according to Dutch guidelines for animal experimentation.

### Nucleic acid extraction and real-time PCR

**Fig. 1:**
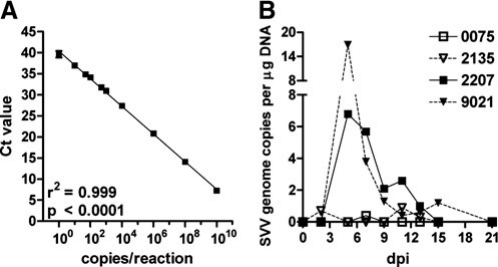
Detection of SVV DNA in blood leukocytes of SVV-infected Chinese rhesus macaques. **a** The efficiency of SVV DNA quatitation was determined in real-time PCR using 1 to 10x10^10^ copies of SVV ORF21 mixed with 100 ng of herring sperm DNA. The SVV ORF21 primer set reliably detected 1 to 10 × 10^10^ copies of SVV DNA (*r* ^2^ = 0.999). **b** SVV DNA levels were determined using real-time PCR with primers specific for SVV ORF 21. All monkeys were positive for SVV DNA, with peak viremia occurring at 5 dpi. SVV DNA copy number varied from very low (monkeys 0075 and 2135) to high (monkeys 2207 and 9021)

DNA was extracted from PBMC, individual or pooled ganglia, and from portions of liver, lung and spleen using the QIAamp DNA Mini Kit (Qiagen, Valencia, CA, USA). Total RNA was isolated from the same tissues using TRIzol reagent (Invitrogen, Carlsbad, CA, USA) and subsequently with the RNeasy Mini Kit (Qiagen). cDNA synthesis was performed as described (Messaoudi et al. 2009) using 1 μg total RNA and Superscript III RT (Invitrogen) with random primers. Quantitative real-time PCR (qPCR) was performed in triplicate on DNA and cDNA using Taqman 2× PCR Universal Master Mix (Applied Biosystems, Foster City, CA, USA) with primers and probes specific for SVV DNA ORFs 21, and ORFs 9, 61, and 63 (cDNA) as described (Messaoudi et al. 2009). SVV DNA amplicons were included in each qPCR assay and yielded a consistent inverse relationship between the Ct value and the amount of input template DNA. The detection limit of the SVV DNA qPCR assay, based on the ORF21 primers/probe set, was 1 copy SVV DNA/μg DNA (Fig. 1a). Single-copy gene oncostatin-M (OSM) and GAPDH (glyceraldehyde-3-phosphate dehydrogenase) were used as endogenous controls for DNA and RNA integrity, respectively, according to the manufacturer’s instructions and as described (Bruce et al. 2005).

### SVV-specific ELISA

Plasma SVV-specific IgG titers were determined by ELISA. Briefly, 96-well flat-bottom plates were coated with protein lysates of mock- and SVV-infected Vero cells (2 μg/ml) overnight at 4°C, washed twice with distilled water, and blocked with 3% bovine serum albumin (BSA) diluted in PBS for 1 h at 37°C. Three-fold dilutions of plasma samples were prepared in PBS containing 1% BSA and incubated for 1 h at 37°C. After 10 washes with 0.05% Tween 20 in PBS, the wells were incubated with horseradish peroxidase (HRP)-conjugated rabbit anti-human IgG (DakoCytomation) diluted in 1% BSA in PBS for 1 h. The plates were washed 10 times and incubated with the substrate 3, 3′, 5, 5′-etramethylbenzidine at room temperature. After 10 min, the reaction was stopped by addition of 2 M sulfuric acid, and optical density (OD) was measured at 405 nm using 620 nm as a reference. End-point titers were obtained by log-log transformation of the linear proportion of the curve using 0.2 OD units as a cut-off.

### Virus neutralization assay

Plasma SVV neutralization IgG titers were determined by plaque reduction assay on Vero cells as described (Soike et al. 1991). Briefly, serial 2-fold dilutions of heat-inactivated macaque plasma samples were incubated with ~100 pfu of SVV for 1 h at room temperature. Plasma samples were plated in duplicate on confluent Vero cells in 6-well plates. After 7 days of culture at 37°C, monolayers were fixed in 4% paraformaldehyde and stained with 0.2% crystal violet solution to visualize virus plaques. The SVV neutralizing titer was expressed as the highest dilution showing an 80% reduction in virus plaques compared to control samples cultured in the absence of plasma.

### Flow cytometry

PBMCs were stained with monoclonal antibodies (mAbs) directed against human CD20 (clone L27; BD Biosciences, San Diego, CA, USA), IgD (Southern Biotech, Birmingham, AL, USA), and CD27 (O323; BD Biosciences) to discriminate between naïve and memory B-cell subsets (Messaoudi et al. 2009; Vugmeyster et al. 2004). T-cell subsets were identified using mAbs directed against human CD3 (SP34-2), CD4 (L200), CD8 (SK1), CD28 (CD28.2), and CD95 (DX2) (all BD Biosciences) to distinguish between naïve, central memory and effector memory T-cells (Messaoudi et al. 2009; Pitcher et al. 2002). Cells were fixed in Cytofix/Cytoperm (BD Biosciences) and stained with antibodies against granzyme B (grB; GB11; BD Biosciences) to detect cytotoxic T-cells. To detect proliferating B- and T-cells, the nuclear membrane was permeabilized using 10% DMSO in Cytofix/Cytoperm (BD Biosciences), and cells were stained with antibody directed against the cell proliferation marker Ki67 (clone B56; BD Biosciences). Fluorescence was detected on a FACS Canto II and analyzed using FACS Diva software (BD Biosciences).

### Detection of SVV-specific T-cells

PBMCs were stimulated overnight with predefined optimal concentrations of protein lysates generated from mock and SVV-infected Vero cells, and mock-infected DBS-FRhL-2 cells, followed by incubation with Golgistop (BD Biosciences) for 6 h at 37°C to block cytokine secretion. After stimulation, cells were stained with mAbs directed against human CD3, CD4, CD8, CD28, and CD95 as described above. Samples were fixed and permeabilized using Cytofix/Cytoperm and incubated with a human interferon (IFN)-γ-specific mAb B27 (BD Biosciences). Fluorescence was measured on a FACS Canto II and analyzed using FACS Diva software.

## Results

### Clinical course of acute SVV infection in Chinese rhesus macaques

To investigate the virus and host immune factors during acute SVV infection, four SVV naïve Chinese rhesus macaques were infected intratracheally with 5 × 10^4^ pfu cell-associated SVV-WT (*n* = 2) or 5 × 10^3^ pfu SVV-GFP (*n* = 2). SVV-GFP was used to enable visualization of SVV-infected cells in blood during viremia and in skin lesions. Physical examinations every other day revealed no skin lesions. All animals developed a viremia. SVV DNA levels were higher in monkeys 2207 and 9021 that received the highest SVV dose, compared to animals 0075 and 2135 that received a lower dose of SVV and had trace levels of SVV DNA in peripheral blood mononuclear cells (PBMCs; Fig. 1b). SVV DNA was detected 2 to 15 dpi and peaked at 5 dpi. No infectious SVV was recovered from PBMCs at any time during viremia, and no SVV- or GFP-positive cells were detected by flow cytometry in whole-blood (data not shown).

### Detection of SVV DNA and RNA in ganglionic and non-ganglionic tissues of SVV-infected rhesus macaques

To determine if SVV ganglionic infection was inhibited by the absence of overt viral replication in skin, the SVV DNA load in dorsal root ganglia and trigeminal ganglia at 21 dpi was determined by qPCR (Table 1). SVV DNA was detected in most pooled ganglia, indicating hematogenous infection. The SVV DNA load was higher in monkeys infected with higher doses of virus (monkeys 2207 and 9021). SVV DNA load in non-ganglionic tissues (i.e., lung, liver, and spleen) was undetectable or lower compared to ganglia of the same monkey (Tables 1 and 2).

**Table 1:** Quantification of SVV-specific DNA and transcripts in ganglia from Chinese rhesus macaques at 21 days post infection

Monkey	Ganglion	DNA target^a^		RNA target^b^			
		ORF 21	OSM^c^	ORF 9	ORF 61	ORF 63	GAPDH^d^
0075	Trigeminal	6	positive^e^	nd^f^	nd	nd	nd
	Cervical	0	positive	nd	nd	nd	nd
	Thoracic	0	positive	nd	nd	nd	nd
	Lumbar	trace^g^	positive	und^h^	3	und	positive
	Sacral	4	positive	und	8	und	positive
2135	Trigeminal	28	positive	nd	nd	nd	nd
	Cervical	8	positive	trace	81	trace	positive
	Thoracic	117	positive	trace	372	6	positive
	Lum/Sac^i^	16	positive	trace	138	trace	positive
2207	Trigeminal	60	positive	nd	nd	nd	nd
	Cervical	2	positive	trace	75	7	positive
	Thoracic	73	positive	nd	nd	nd	nd
	Lumbar	99	positive	und	15	trace	positive
	Sacral	38	positive	nd	nd	nd	nd
9021	Trigeminal	860	positive	nd	nd	nd	nd
	Cervical	230	positive	und	582	trace	positive
	Thoracic	687	positive	nd	nd	nd	nd
	Lumbar	698	positive	trace	692	trace	positive
	Sacral	64	positive	9	8089	239	positive

^a^SVV genome copies/ug of total DNA

^b^SVV transcript copies/μg of total RNA.

^c^Oncostatin-M

^d^Glyceraldehyde 3-phosphate dehydrogenase.

^e^Specific amplicon detected.

^f^Not done

^g^>2 copies of SVV DNA or cDNA in 1 or 2 out of 3 reactions

^h^SVV DNA or transcript undetectable.

^i^Pooled lumbar and sacral ganglia

**Table 2:** Quantification of SVV-specific DNA and transcripts in non-ganglionic tissues from Chinese rhesus macaques at 21 days post infection

Monkey	Tissue	DNA PCR^a^		cDNA PCR^b^			
		ORF 21	OSM^c^	ORF 9	ORF 61	ORF 63	GAPDH^d^
0075	Lung	3	positive^e^	und^f^	und	und	positive
	Liver	und	positive	und	und	und	positive
	Spleen	und	positive	und	und	und	positive
2135	Lung	und	positive	und	und	und	positive
	Liver	und	positive	und	und	und	positive
	Spleen	und	positive	und	und	und	positive
2207	Lung	und	positive	und	und	und	positive
	Liver	und	positive	und	und	und	positive
	Spleen	und	positive	und	und	und	positive
9021	Lung	und	positive	und	und	und	positive
	Liver	3	positive	und	und	und	positive
	Spleen	1	positive	und	und	und	positive

^a^SVV genome copies/ug of total DNA

^b^SVV transcript copies/μg of total RNA

^c^Oncostatin-M.

^d^Glyceraldehyde 3-phosphate dehydrogenase.

^e^Specific amplicon detected

^f^SVV DNA or transcript undetectable

Like VZV latency in humans, SVV latency is associated with the restricted transcription of several immediate early and early transcripts (Kennedy and Cohrs 2010; Kennedy et al. 2004). Levels of the SVV latency-associated ORF 61 and ORF 63 transcripts, as well as the late SVV ORF 9 transcript were determined in ganglia and non-ganglionic tissues of all monkeys. No SVV transcripts were detected in lung, liver and spleen (Table 2). SVV ORF 61 was the most prevalent and abundant SVV transcript found in ganglia (Table 1). Lower levels of SVV ORF 63 transcripts were detected in ganglia from all but one monkey (0075) that also had a low SVV DNA load. Trace levels of SVV ORF 9 transcripts were detected in ganglia of 3 of 4 monkeys. The detection of low levels of the late SVV gene ORF 9 transcript, along with higher levels of SVV latency-associated transcripts in monkey 9021 indicated that latency was not completely established at 21 dpi.

### B-cell response in SVV-infected rhesus macaques

**Fig. 2:**
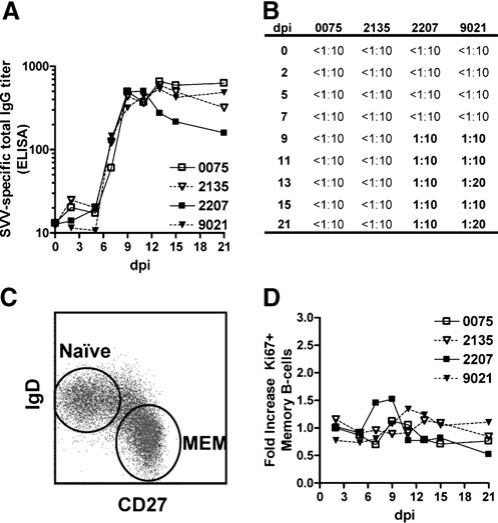
Induction of SVV-specific B-cell response in infected Chinese rhesus macaques. **a** SVV-infected macaques developed a SVV-specific plasma IgG response starting at 7 dpi, peaking at 9 to 11 dpi and remaining high until 21 dpi as determined by SVV ELISA. **b** SVV neutralizing antibodies were detected in animals 2207 and 9021 starting at 9 dpi. **c** Peripheral blood B-cells identified by flow cytometry based on expression of CD20 were further distinguished as naïve (IgD^+^CD27^−^) or memory (MEM; IgD^−^CD27^+^) B-cells. **d** Proliferation of memory B-cells as assessed by flow cytometry based on expression of the cell proliferation marker Ki67 and given as fold-increase in Ki67+ cells compared to the sample obtained at 0 dpi

All monkeys had detectable plasma SVV-specific IgG titers at 7 dpi, which peaked between 9 and 11 dpi and remained high until the end of the 21-day study period (Fig. 2a). Comparable levels of SVV-specific plasma IgG titers were found in all monkeys and monkeys 2207 and 9021 developed virus-neutralizing antibody titers ≥1:10 (Fig. 2b).

Upon antigen recognition, antigen-specific B-cells proliferate and mature into isotype-switched memory B-cells and plasma cells (Srivastava et al. 2005). To identify proliferating B-cell subsets, CD20^+^ cells were divided into naïve (IgD^+^CD27^−^) and memory (IgD^−^CD27^+^) B-cells using flow cytometry (Fig. 2c) and stained for the proliferation marker Ki67 (Scholzen and Gerdes 2000; Vugmeyster et al. 2004). Compared to 0 dpi, only a marginal increase (1.2- to 1.5-fold) in proliferating memory B-cells was seen in 3 of 4 monkeys from 9 to 11 dpi (Fig. 2d).

### T-cell response in SVV-infected rhesus macaques

**Fig. 3:**
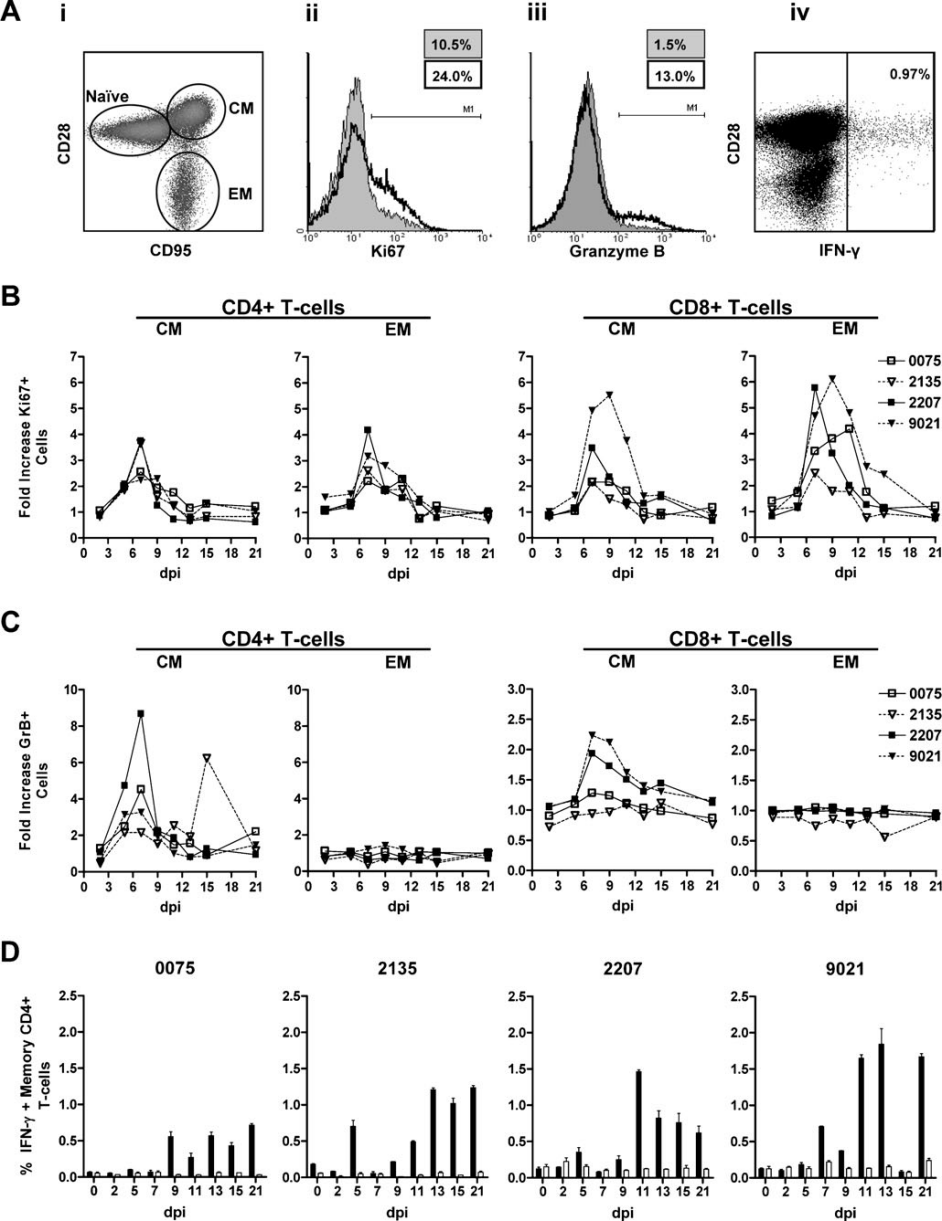
Induction of systemic SVV-specific T-cell response in infected Chinese rhesus macaques. **a** *i* Peripheral blood T-cells were identified based on expression of CD3 and categorized further based on expression of CD28 and CD95 as naïve (CD28^+^CD95^-^), central memory (CM; CD28^+^CD95^+^) and effector memory (EM; CD28^−^CD95^+^) T-cells. **a** *ii* Proliferating T-cells were identified by flow cytometric detection of cell proliferation marker Ki67, shown for CM CD4^+^ T-cells from animal 9021 at 0 (*filled area*) and 9 (*black line*) dpi. **a** *iii* Granzyme B expression in CM CD4^+^ T-cells from animal 2207 at 0 (*filled area*) and 7 (*black line*) dpi. **a** *iv* SVV-specific IFN-γ-producing memory CD4^+^ T-cells from animal 9021 at 21 dpi. **b** Kinetics of proliferation in T-cell subsets, with a peak in proliferation of CM and EM CD4^+^ at 7 dpi and peak proliferation in CM and EM CD8^+^ T-cells at 7 to 9 dpi and 7 to 11 dpi, respectively. **c** Kinetics of granzyme B expression in T-cell subsets, showing a relative increase in CM CD4^+^ and CD8^+^ at 7 dpi. **d** Percentage of IFN-γ producing peripheral blood memory CD4^+^ T-cells in response to protein lysates prepared from mock- and SVV- infected Vero cells (netto responses are shown in *black bars*) and mock-infected rhesus macaque FRhL-2 cells (*white bars*)

Primary VZV infection in humans induces a profound T-cell mediated immune response, which occurs after the onset of varicella skin rash and is essential in controlling viremia (Weinberg and Levin 2010). Upon recognition of the cognate antigen, T-cells proliferate and mature to memory T-cells. To assess T-cell expansion in SVV-infected rhesus macaques, blood-derived T-cells (CD3^+^ cells) were differentiated into naïve (CD28^+^CD95^−^), central memory (CD28^+^CD95^+^) and effector memory (CD28^−^CD95^+^; Pitcher et al. 2002) and stained for Ki67 (Fig. 3ai, ii). Intratracheal inoculation with cell-associated SVV induced proliferation of CD4^+^ and CD8^+^ T-cells in the central and effector memory compartments of all rhesus macaques at 7 dpi (Fig. 3b). More proliferation was induced in CD8^+^ compared to CD4^+^ T-cells, and more proliferating CD8^+^ T-cells were found in monkeys that received the highest virus dose (monkeys 2207 and 9021). VZV infection also induces virus-specific cytotoxic CD4^+^ and CD8^+^ T-cells that express granzyme B (grB) and secrete soluble mediators such as IFN-γ (Hayward et al. 1996; Malavige et al. 2007). Intratracheal inoculation with cell-associated SVV resulted in an increase in grB-expressing CD4^+^ and CD8^+^ central but not effector memory T-cells, with the highest numbers seen at 7 dpi; induction of grB^+^CD8^+^ central memory T-cells was found only in monkeys 2207 and 9021 (Fig. 3c).

The Ki67 and grB response shown in Fig. 3b, c might have been induced by either viral proteins or allogeneic major histocompatibility complex (MHC) proteins expressed by uninfected DBS-FRhL-2 cells used to generate the SVV inoculum. Thus, the frequency of antigen-specific T-cells was determined by intracellular staining for IFN-γ at all time points in all monkeys using various control cell lysates (Fig. 3aiv, d). First, we included protein lysates of mock- and SVV-infected Vero cells to differentiate T-cell reactivity directed against SVV antigens. Second, T-cell reactivity directed towards proteins lysates of mock-infected DBS-FRhL-2 cells was assayed to detect the induction of allo-MHC specific T-cell responses after intratracheal inoculation of cell-associated SVV in rhesus macaques. While no MHC response was detected, the SVV-specific memory CD4+ T-cell response was detected starting at 9 dpi (Fig. 3d). Analogous to the frequency of grB + T-cells, most IFN-γ-secreting CD4^+^ T-cells had a central memory phenotype (Fig. 3aiv data not shown) as was also seen in grB^+^ T-cells (Fig. 3c). Overall, all monkeys developed a virus-specific CD4 T-cell immune response beginning about 4 days after the peak in viremia.

## Discussion

The present study showed that after primary infection with SVV, Chinese rhesus macaques developed viremia, ganglionic infection by the hematogenous route and virus-specific memory B- and T-cell responses in the absence of rash. The absence of rash contrasts with previous studies, which showed that inoculation of Indian rhesus macaques deep into the bronchial tree with similar virus loads of the same SVV strain lead to viremia, SVV-specific adaptive immune responses and varicella rash at 7–10 dpi (Mahalingam et al. 2010; Messaoudi et al. 2009). While both the trachea and bronchi harbor cells susceptible to SVV infection, the anatomic location within the respiratory tract at which the virus is administrated might affect initial virus replication. Alternatively, the geographic origin of rhesus macaques may affect their susceptibility to SVV infection. Whole-genome sequencing has revealed extensive genetic differences in rhesus macaques of Indian and Chinese origin (Gibbs et al. 2007; Hernandez et al. 2007) that are likely to play a role in the different disease progression and host response seen after SIV infection (Ling et al. 2002; Trichel et al. 2002).

VZV in humans is thought to enter ganglia either via hematogenous transport within infected lymphocytes or by retrograde axonal transport from varicella skin lesions (Annunziato et al. 2000; Ozaki et al. 1994). The latter notion is based largely on studies with herpes simplex virus, while only indirect evidence exists for VZV. For example, VZV ORF29 protein is expressed in Schwann cells and axons of nerves in varicella, but not in zoster lesions (Annunziato et al. 2000). Also, VZV can infect axons and undergo retrograde transport to neuronal cell bodies in vitro (Markus et al. 2011). The data presented herein reveal that SVV replication in the skin of intratracheally infected rhesus macaques is not a prerequisite for virus to infect ganglia. The association between SVV DNA loads in blood and ganglia further supports the role of SVV-infected lymphocytes in the establishment of ganglionic infection. Similarly, the high correlation of VZV seropositivity with the presence of VZV DNA in ganglia (Verjans et al. 2007) indicates subclinical hematogenous infection in VZV seropositive humans without a history of chickenpox.

Compared to SVV infection of Indian rhesus macaques (Messaoudi et al. 2009), a dampened adaptive B- and T-cell response, with reduced peak endpoint SVV-specific IgG titers, a lower proliferative B-cell response and a lower frequency of proliferating T-cells and SVV-specific T-cells were seen after SVV infection of Chinese rhesus macaques. Note that live attenuated VZV Oka vaccine typically does not cause varicella rash in humans and elicits an immune response that is less robust than that seen after natural VZV infection (Watson 2008; Weinberg et al. 2010), although it is protective (Hayward et al. 1996; Levin et al. 1994; Takahashi et al. 1974). The kinetics of the T-cell response found herein is similar to that found by Messaoudi et al. (2009), who reported a peak in T-cell proliferation and grB expression shortly after peak viremia in macaques. However, the SVV-specific immune response herein was dominated by T-cells exhibiting a central memory rather than an effector memory phenotype, consistent with studies on the phenotype of VZV-specific T-cells in humans which express the co-stimulatory molecule CD28 and the lymph node-homing receptors CCR7 and CD62L (Malavige et al. 2008a, b). The marked similarities between SVV infection in rhesus macaques and humans vaccinated with the varicella vaccine emphasize the applicability of this animal model to develop novel therapeutic strategies against VZV infection.

Overall, our results demonstrate that acute SVV infection of Chinese rhesus macaques leads to ganglionic infection and the induction of a virus-specific adaptive B- and T-cell memory response in the absence of skin rash. Importantly, some humans have serum antibody to VZV without any history of varicella. Yet VZV is latent in ganglia of nearly all humans. A logical interpretation of these facts is that human ganglia are infected hematogenously, a notion supported by our demonstration that ganglia of Chinese rhesus macaques are infected hematogenously by SVV.
